# Nest Microbial Community Dynamics Within the Nest‐Building Stage of Green‐Backed Tits ( *Parus monticolus* )

**DOI:** 10.1002/ece3.72124

**Published:** 2025-09-07

**Authors:** Nan Yang, Jiajia Xin, Qun Tu, Xiaoyang Bao, Haibo Zhang, Yijiang Su, Xiongwei Yang, Canshi Hu

**Affiliations:** ^1^ College of Life Sciences Guizhou University Guiyang Guizhou China; ^2^ Aha Lake National Wetland Park Guiyang Guizhou China; ^3^ Research Center for Biodiversity and Nature Conservation Guizhou University Guiyang Guizhou China

**Keywords:** artificial nest boxes, co‐occurrence networks, green‐backed tits, nest microbiota

## Abstract

The microbiota within bird nests is considered an important factor influencing the reproductive processes of birds. Certain pathogenic microorganisms present in nest environments may compromise avian health through direct infection of both adult birds and their offspring, ultimately leading to reduced reproductive success. However, there is a lack of systematic research on the changes in the microbial environment within the nest during the nest‐building stages, where nest‐building activities may exert a positive impact on the associated microbial communities. Here, diversity, microbial composition, and co‐occurrence network in the nests of green‐backed tits ( *Parus monticolus* ) during both pre‐ and post‐nest‐building stages were investigated using high‐throughput sequencing technology coupled with microbial network analysis. We found that the nests had significant differences in the microbial communities between the pre‐ and post‐nest‐building stages of green‐backed tits. The microbial community in the post‐nest‐building stage of green‐backed tits was predominantly composed of beneficial bacteria, exhibiting an overall trend of increased abundance of beneficial microorganisms and decreased prevalence of pathogenic microorganisms. Furthermore, the complexity of the microbial co‐occurrence networks decreased, whereas their stability was enhanced in the post‐nest‐building stage of green‐backed tits. These findings highlight the impact of green‐backed tits on the microbial community within the nest, revealing that the microecology becomes healthier and more stable during the post‐nest‐building stage of green‐backed tits, and will help us gain a deeper understanding of the interactions between avian species and nest microbiota.

## Graphical abstract

The microbiota in bird nests significantly influences avian reproduction. Using high‐throughput sequencing and microbial network analysis, we examined the microbial diversity, composition, and co‐occurrence networks in green‐backed tit ( *Parus monticolus* ) nests during pre‐ and post‐nesting stages. Post‐nesting nests showed increased beneficial microbes, reduced pathogens, and more stable microbial networks, indicating a healthier microecology and highlighting the interaction between birds and nest microbiota.

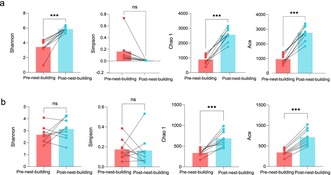

## 1 Introduction

The living environment plays a crucial role in animal survival and reproduction. Particularly for breeding individuals, a stable and healthy microenvironment is essential to ensure successful reproductive activities, with nests serving as critical habitats that fulfill these requirements (Chung et al. 2023). Nests are indispensable places for egg‐laying and incubation in Ovipara, helping to protect Ovipara from the weather and natural enemies (Martin 2017). Nests can be primarily categorized into two distinct types based on structural characteristics: open nests and enclosed nests (Barros‐Leite and Francisco 2024). Enclosed nest environments, such as cavity nests, create microhabitats that are particularly conducive to microbial colonization and proliferation due to the relatively stable microclimatic conditions within the nests (Hanzelka et al. 2023). Distinct from the ambient environmental microbiota, the microbial communities within animal nests play a pivotal role in influencing the health and developmental outcomes of both the nesting animals and their offspring (Lindström et al. 2021; Goodenough and Stallwood 2012; Brandl et al. 2014). Pathogenic microorganisms in the nest not only directly endanger the health of the animal, but also indirectly by infesting the gut. For example, the presence of genera *Pseudomonas* and *Aeromonas* in nesting sea turtles ( *Dermochelys coriacea* ) may affect hatching success rates, particularly during early incubation (Soslau et al. 2011). The presence of genera *Paenibacillus*, *Sporosarcina*, and *Bacillus* in red mason bee ( *Osmia bicornis* ) nests leads to larval mortality through two primary mechanisms: direct toxin exposure and intestinal tract infection (Voulgari‐Kokota et al. 2020). However, the microbial community within nests also improves the nest environment. Certain microbes that produce antimicrobial metabolites have established symbiotic relationships with social insects such as wasps and termites to prevent infection by pathogens (Schmidt et al. 2022; Turillazzi et al. 2023; Chavarría‐Pizarro et al. 2024). Therefore, studying nest microbiota could enhance our comprehension of the interactions between these microbial communities and their animal hosts, thereby advancing species conservation initiatives.

For birds, the consistent physical conditions within the nest offer a distinctive habitat for microbiota, prompting many to establish colonization within the nest (Burtt Jr. and Ichida 1999). Research indicates that microbial communities mediate the entire life history of birds, transferring among nest materials, adult birds, and chicks, and collectively influencing avian reproductive development (Pugh 1967). Some potentially pathogenic microorganisms will persist in nests for extended periods. When pathogens dominate, it causes a decline in the animals' physical condition, making them more susceptible to competition and predation (Goodenough and Stallwood 2012; Brandl et al. 2014). In cases of severe infection, this will result in death (Nuttall 1997). Particularly, *Aspergillus fumigatus* , deemed one of the most dangerous fungal pathogens to birds, infects the avian lungs, causing pulmonary aspergillosis, and also permeating through eggshells, leading to the death of the embryos (Korniłłowicz‐Kowalska and Kitowski 2013). *Pseudomonas aeruginosa* is capable of breaching eggshells, leading to the death of embryos, and also inducing sinusitis and conjunctivitis in adult birds (Silvanose et al. 2001; Carter 1982). However, the microbiota also benefits birds by inhibiting pathogens and promoting their growth and development. For example, some members of the *Enterococcus* compete with other pathogenic bacteria, such as *Enterococcus faecalis* , to suppress their pathogenic effects on hatched chicks (Audisio et al. 2000). In studies on the pied flycatcher ( *Ficedula hypoleuca* ), researchers found that *Enterococcus faecium* competitively interacts with *Enterococcus faecalis* to improve the acclimatization of chicks (Moreno et al. 2003). This beneficial bacterium has even been used as a growth promoter for chicks (Moreno et al. 2003).

The early‐life environment plays a critical role in shaping the microbiota of animals, with profound implications for their health, development, and survival (Pereira et al. 2024). Studies have demonstrated that the first environmental exposure in an animal's life influences the establishment and composition of its microbiota (Bokulich et al. 2016; Basso et al. 2022). For birds, the nest represents the earliest and most critical environmental exposure, serving as the primary source of microbial colonization for chicks (Pereira et al. 2024; Chen et al. 2020; Diez‐Méndez et al. 2023; Campos‐Cerda and Bohannan 2020; Martínez‐García et al. 2016). The microbiota within the nest can directly or indirectly influence the microbial communities of the chicks, potentially affecting their growth, immune system development, and overall fitness (Pereira et al. 2024; Chen et al. 2020; Diez‐Méndez et al. 2023; Campos‐Cerda and Bohannan 2020; Martínez‐García et al. 2016). Studies have shown that microbiota may mediate the survival rate of eggs and chicks by affecting the nesting process of birds (Cook et al. 2005), and also enter the chicks' gut through food supply, thereby impacting the chicks' health (Pereira et al. 2024; Chen et al. 2020). In addition, parent birds frequently come into contact with microbiota in the nest during the processes of nest building and chick rearing. Microbiota (such as *Cladosporium cladosporioides* and *Pseudomonas aeruginosa* ) may enter the parent birds' bodies through their skin or respiratory tracts, potentially affecting their immune status and physiological health (Silvanose et al. 2001; Carter 1982; Kanj et al. 2023; Lee et al. 2025). Therefore, to ensure the health of both the parent birds and their chicks, parent birds may actively regulate the composition and structure of the nest's microbial community during the nesting process, maintaining the health and stability of the nest's microbiota (Ruiz‐Castellano et al. 2016). Based on this, we intend to verify this hypothesis by exploring the dynamic changes in the microbial community during the pre‐ and post‐nest‐building stage. We employed high‐throughput sequencing technology and microbial network analysis to evaluate the impact in the post‐nest‐building stage of green‐backed tits ( *Parus monticolus* ) on the diversity, community structure, and co‐occurrence patterns of nest microbiota. We selected the green‐backed tits as a study subject because it is a typical secondary cavity‐nesting bird that is widely distributed in China and is well represented. By focusing on the post‐nest‐building stage, we aim to investigate how the green‐backed tits influence the microbial environment of its nest after nesting. We hypothesize that: (i) The diversity and composition of microbial communities within green‐backed tits nests may change following nest construction. To enhance reproductive success, we predict an increase in beneficial microorganisms and a corresponding decrease in pathogenic microorganisms within the nest environment. (ii) Changes in microbial diversity and composition may lead to alterations in the interactions between microbial communities.

## 2 Results

### 2.1 Sample Sequencing Analysis

**FIGURE 1:**
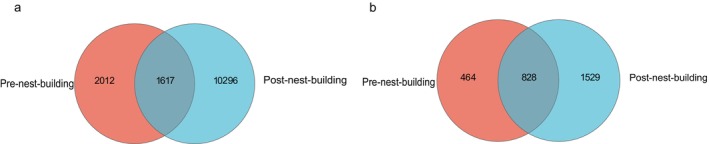
The Venn diagram of bacteria (a) and fungi (b) in the pre‐ and post‐nest‐building stages of green‐backed tits.

The microbial composition of green‐backed tits nests based on 16S rRNA and ITS sequencing technology was analyzed. According to the sequencing results, a total of 1,356,320 optimized sequences were obtained from the 18 bird nest bacterial samples, with an average sequence length of 376 bp. A total of 1,235,310 optimized sequences were obtained from 18 bird nest fungal samples, with an average sequence length of 249 bp. The number of OTUs shared by pre‐ and post‐nest‐building stages were 1617 and 828 respectively in bacteria and fungi (Figure 1a,b).

### 2.2 Microbial Diversity Analysis in the Pre‐ and Post‐Nest‐Building Stages of Green‐Backed Tits

**FIGURE 2:**
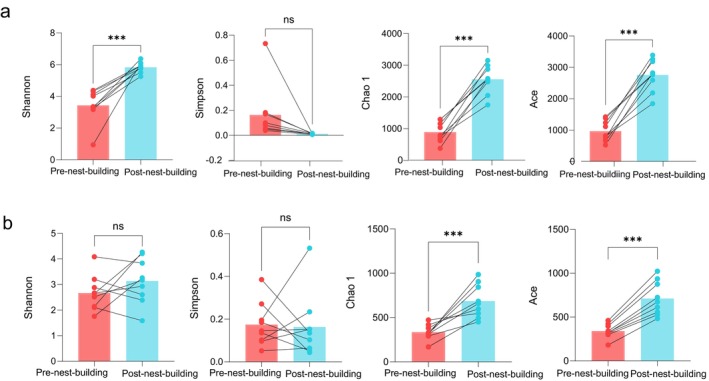
Paired Samples t‐test of bacterial (a) and fungal (b) diversity in the pre‐ and post‐nest‐building stages of green‐backed tits. *p < 0.05 indicates that the results are statistically significant; **p < 0.01 indicates that the results are highly significant; ***p < 0.001 indicates that the results are extremely significant; ns indicates statistically insignificant.

The post‐nest‐building stage of green‐backed tits had a significant effect on the diversity indices of both bacterial and fungal communities (Figure 2). For bacteria, Shannon, Chao1, and Ace indices were significantly higher in nests during the post‐nest‐building stage compared to the pre‐nest‐building stage of green‐backed tits (Figure 2a). For fungi, Chao1 and Ace indices were significantly higher in nests during the post‐nest‐building stage compared to the pre‐nest‐building stage of green‐backed tits (Figure 2b). However, the Simpson index showed no significant changes in either bacterial or fungal communities between the pre‐ and post‐nest‐building stages of green‐backed tits.

**FIGURE 3:**
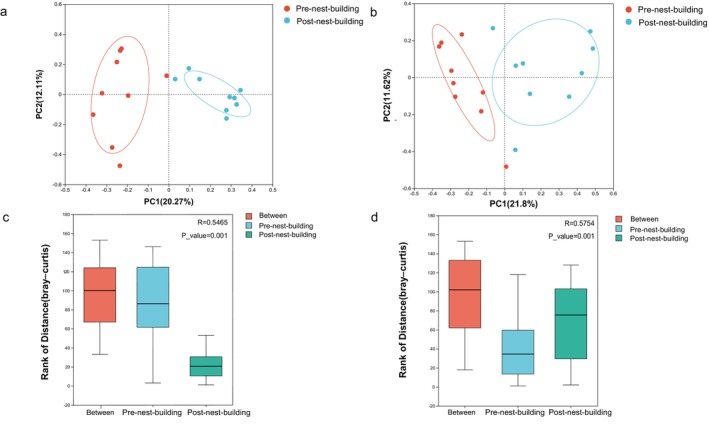
PCoA cluster analysis and ANOSIM analysis of microbial community structure in the pre‐ and post‐nest‐building stages of green‐backed tits. (a, c) PCoA cluster analysis and ANOSIM analysis of bacteria. (b, d) PCoA cluster analysis and ANOSIM analysis of fungi. An R value closer to 1 indicates stronger inter‐group than intra‐group dissimilarity, while values approaching 0 suggest no meaningful separation. *p* < 0.05 indicates that the results are statistically significant; *p* < 0.01 indicates that the results are highly significant; *p* < 0.001 indicates that the results are extremely significant.

PCoA demonstrated pronounced clustering of nest microbial communities, exhibiting strong between‐group aggregation and within‐group segregation when comparing the pre‐ and post‐nest‐building stages, which suggests notable dissimilarities in microbial structure. Furthermore, ANOSIM analysis revealed that between‐group differences were significantly greater than within‐group variations (Figure 3c,d *p* < 0.05), demonstrating significant differences in microbial communities between the pre‐ and post‐nest‐building stages.

### 2.3 Microbial Community Composition in the Pre‐ and Post‐Nest‐Building Stages of Green‐Backed Tits

**FIGURE 4:**
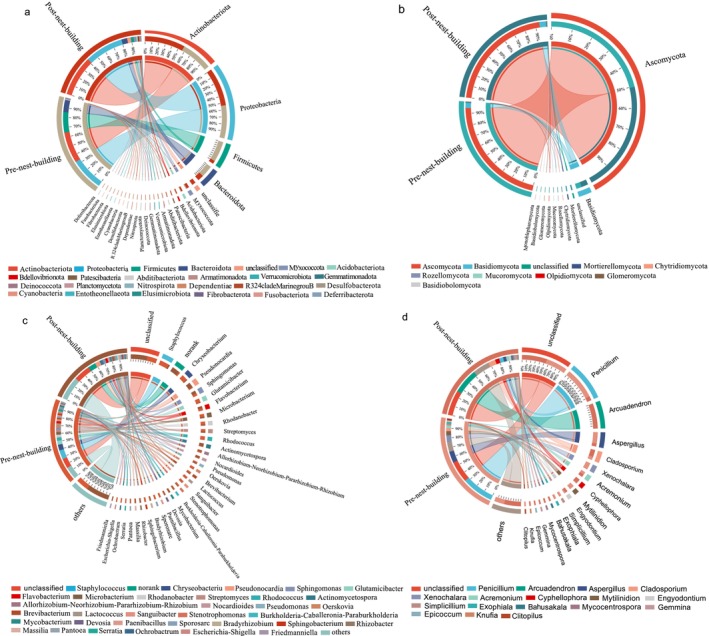
Microbial community composition in the pre‐ and post‐nest‐building stages of green‐backed tits. Composition of bacteria (a) and fungi (b) at the phylum level, with figures shown all the phyla in the pre‐ and post‐nest‐building stages of green‐backed tits. Composition of bacteria (c) and fungi (d) at the genus level, only the dominant genera (greater than 1% abundance) in the pre‐ and post‐nest‐building stages of green‐backed tits. Unclassified and norank and relative abundance less than 1% were grouped into separate categories in the figure.

**FIGURE 5:**
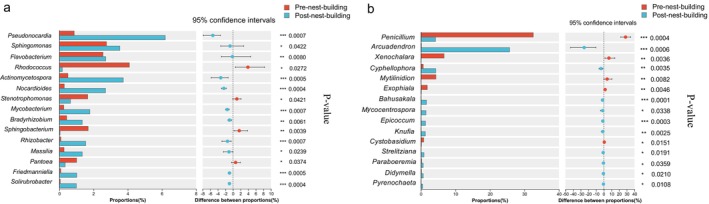
Differences in bacteria (a) and fungi (b) composition at the genus level between the top 15 taxa in the pre‐ and post‐nest‐building stages of green‐backed tits. *p < 0.05 indicates that the results are statistically significant; **p < 0.01 indicates that the results are highly significant; ***p < 0.001 indicates that the results are extremely significant.

According to taxonomic analysis, a total of 25 phyla were identified in the bacterial community at the phylum level (Figure 4a). In the pre‐nest‐building stage of green‐backed tits, Proteobacteria (33.43%), Actinomycetota (31.50%), Firmicutes (20.79%) and Bacteroidota (12.18%) were the dominant bacteria. Actinomycetota (41.18%), Proteobacteria (39.03%), Bacteroidota (5.76%), Chloroflexi (3.68%), Firmicutes (2.91%) and Myxococcota (1.53%) were the dominant bacteria in the post‐nest‐building stage of green‐backed tits. At the genus level, a total of 481 bacteria were identified in the bacterial community, with 24 dominant bacteria in the pre‐nest‐building stage of green‐backed tits and 18 dominant bacteria in the post‐nest‐building nests of green‐backed tits (Figure 4c). In the pre‐nest‐building stage of green‐backed tits, *Staphylococcus* (11.29%), *Chryseobacterium* (7.15%), *Glutamicibacter* (4.81%), *Rhodococcus* (4.09%), and *Rhodanobacter* (3.48%) were the top 5 dominant bacteria. *Pseudonocardia* (6.19%), *Actinomycetospora* (3.73%), *Sphingomonas* (3.54%), *Flavobacterium* (2.71%), and *Nocardioides* (2.70%) were the top 5 dominant bacteria in the post‐nest‐building nests of green‐backed tits. The bacterial composition of the first 15 taxa was compared at the genus level between the pre‐ and post‐nest‐building stages of green‐backed tits. We found that these 15 genera showed significant differences between the pre‐ and post‐nest‐building stages of green‐backed tits. Compared with the pre‐nest‐building stage of green‐backed tits, the relative abundance of 11 genera, including *Pseudonocardia*, *Sphingomonas*, *Flavobacterium*, *Actinomycetospora*, *Nocardioides*, *Mycobacterium*, *Bradyrhizobium*, *Rhizobacter*, *Solirubrobacter*, *Friedmanniella*, and *Massilia* was significantly higher in the post‐nest‐building stage of green‐backed tits (*p* < 0.05). The relative abundance of *Rhodococcus*, *Stenotrophomonas*, *Sphingobacterium*, and *Pantoea* was significantly lower in nests during the post‐nest‐building stage compared to the pre‐nest‐building stage of green‐backed tits (Figure 5a). The fungi of those samples were classified into 10 phyla according to taxonomic analysis (Figure 4b). Both Ascomycota and Basidiomycota were the main components in the pre‐ and post‐nest‐building stages of green‐backed tits, but they differed in relative abundance. The relative abundance in the pre‐nest‐building stage of green‐backed tits was Ascomycota (94.73%) and Basidiomycota (4.37%). The relative abundance in the post‐nest‐building stage of green‐backed tits was Ascomycota (93.44%) and Basidiomycota (5.73%). At the genus level, a total of 559 fungi were identified in the fungal community, with 10 dominant fungi in the pre‐nest‐building stage of green‐backed tits and 12 dominant fungi in the post‐nest‐building stage of green‐backed tits (Figure 4d). In the pre‐nest‐building stage of green‐backed tits, *Penicillium* (32.55%), *Aspergillus* (11.55%), *Xenochalara* (6.69%), *Mytilinidion* (4.34%), and *Cladosporium* (3.09%) were the top 5 dominant fungi. *Arcuadendron* (25.68%), *Cladosporium* (7.33%), *Cyphellophora* (4.27%), *Penicillium* (4.22%), and *Aspergillus* (2.95%) were the top 5 dominant fungi in the post‐nest‐building stage of green‐backed tits. The fungal composition of the first 15 taxa was compared at the genus level between the pre‐ and post‐nest‐building stages of green‐backed tits. We found that these 15 genera showed significant differences between the pre‐ and post‐nest‐building stages of green‐backed tits. Compared with the pre‐nest‐building stage of green‐backed tits, the relative abundance of 10 genera, including *Arcuadendron, Cyphellophora, Bahusakala, Mycocentrospora, Epicoccum, Knufia, Strelitziana, Paraboeremia, Didymella*, and *Pyrenochaeta* was significantly higher in the post‐nest‐building stage of green‐backed tits (*p* < 0.05). The relative abundance of *Penicillium, Xenochalara, Mytilinidion, Exophiala*, and *Cystobasidium* was significantly lower in the post‐nest‐building stage of green‐backed tits than in the pre‐nest‐building stage of green‐backed tits (Figure 5b).

### 2.4 Community Difference Analysis in the Pre‐ and Post‐Nest‐Building Stages of Green‐Backed Tits

**FIGURE 6:**
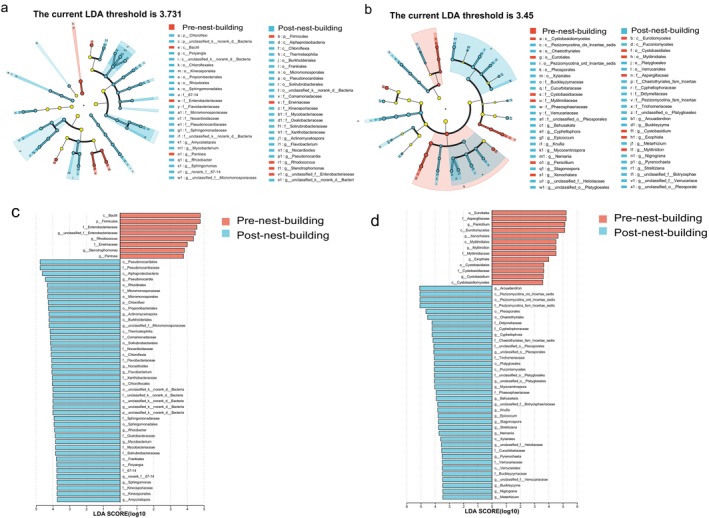
Microbial LEfSe Analysis in the pre‐ and post‐nest‐building stages of green‐backed tits The species differences at different taxonomic levels from phylum to genus (from inner to outer circle) for bacterial communities (a, c) and fungal communities (b, d) are shown respectively.

To identify the biomarkers with statistical differences in nest microbiota between different groups, we performed LEfSe (Linear Discriminant Analysis Effect Size) analysis in the pre‐ and post‐nest‐building stages of green‐backed tits (Figure 6). LDA (Linear Discriminant Analysis) results showed a total of 45 significant biomarkers associated with bacteria, in addition to those unclassified at the phylum level (LDA > 3.731) (Figure 6c). Among them, 8 biomarkers were found in the pre‐nest‐building stage of green‐backed tits, distributed in Firmicutes (2 biomarkers), Proteobacteria (5 biomarkers), and Actinomycetota (1 biomarker). Thirty‐seven biomarkers were found in the post‐nest‐building stage of green‐backed tits, distributed among Proteobacteria (10 biomarkers), Actinomycetota (21 biomarkers), Chloroflexi (3 biomarkers), Bacteroidetes (2 biomarkers), and Myxococcota (1 biomarker). The results related to fungi revealed that there were 13 biomarkers in the pre‐nest‐building stage of green‐backed tits and 37 biomarkers in the post‐nest‐building stage of green‐backed tits (LDA > 3.450) (Figure 6d). The major microbiota were Ascomycota (9 in pre‐nest‐building stage and 31 in post‐nest‐building stage) and Basidiomycota (4 in pre‐nest‐building stage and 6 in post‐nest‐building stage) (Figure 6d). Then, the diagrams of taxonomic clade were made to identify the major microflora. As shown in the cladogram, biomarkers at different taxonomic levels differed significantly between the pre‐ and post‐nest‐building stages of green‐backed tits (Figure 6a,b).

### 2.5 Nest Microbial Co‐Occurrence Network

**FIGURE 7:**
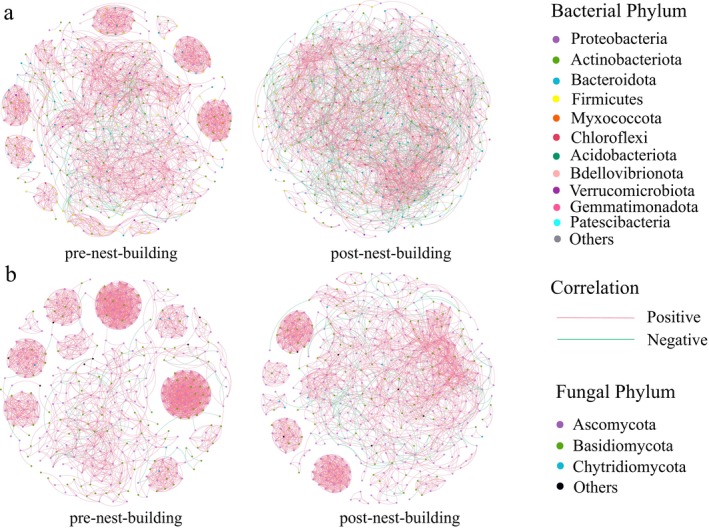
The bacterial (a) and fungal (b) microbial co‐occurrence network in the pre‐ and post‐nest‐building stages of green‐backed tits.

Co‐occurrence networks were used to further investigate the distribution characteristics of nest microbiota in the pre‐ and post‐nest‐building stages of green‐backed tits (Figure 7, Table 1). The results indicated that the bacterial and fungal co‐occurrence networks within the nest exhibited distinct variations between the pre‐ and post‐nest‐building stages of green‐backed tits. Specifically, in the post‐nest‐building stage, both bacterial and fungal co‐occurrence networks displayed a lower number of edges, average degree, clustering coefficient, density, modularity, and percentage of positive edges compared to the pre‐nest‐building stage. However, the post‐nest‐building stage exhibited a higher number of nodes and a higher percentage of negative edges. Changes in these network properties suggested that the network complexity decreased with degradation.

**TABLE 1:** Topological characteristics parameters of microbial co‐occurrence networks.

	Bacteria		Fungi	
	Pre‐nest‐building	Post‐nest‐building	Pre‐nest‐building	Post‐nest‐building
Nodes	487	491	413	493
Edges	2769	2691	2737	2423
Positive edges percent	94.330	75.010	98.210	93.890
Negative edges percent	5.670	24.990	1.790	6.110
Average degree	11.370	10.595	13.254	9.810
Clustering coefficient	0.620	0.386	0.756	0.583
Density	0.023	0.022	0.032	0.020
Modularity	0.812	0.609	0.848	0.775

## 3 Discussion

### 3.1 Changes in the Bacterial Composition in the Post‐Nest‐Building Stage of Green‐Backed Tits

The bacteria that colonize nests may indeed be symbiotic or commensal, aiding in food digestion and complementing the immune system (Devaynes et al. 2018). However, some bacteria may be pathogenic, potentially affecting the growth and survival of chicks. In our study, the diversity of bacterial communities was significantly higher in the post‐nest‐building stage than in the pre‐nest‐building stage of green‐backed tits (*p* < 0.001).

At the phylum level, the dominant bacteria were mainly Proteobacteria (33.43%), Actinomycetota (31.50%), Firmicutes (20.79%) and Bacteroidota (12.18%) in the pre‐nest‐building stage of green‐backed tits, while the dominant bacteria were mainly Actinomycetota (41.18%), Proteobacteria (39.03%), Bacteroidota (5.76%), Chloroflexi (3.68%), Firmicutes (2.91%) and Myxococcota (1.53%) in the post‐nest‐building stage of green‐backed tits. The result was in agreement with studies on the nest microbiota of the Japanese tits ( *Parus minor* ), which belong to the same family (Xin et al. 2023). Among these bacteria, Actinomycetota and Myxococcota are believed to be rich sources of microbial secondary metabolites, which have significant antibacterial activity and will inhibit the invasion of pathogens (Herrmann et al. 2017; Binda et al. 2018; Kachor et al. 2024). Comparing to the pre‐nest‐building stage of green‐backed tits, the relative abundance of Actinomycetota and Myxococcota was higher in the post‐nest‐building stage. Other bacteria, such as Proteobacteria, are often used as a marker of microbial imbalance. They can disrupt the microbial balance of an animal by causing inflammation, respiratory disease, and other ways, leading to animal illness (Kumar and Kumar 2022; Guan et al. 2018). However, Proteobacteria abundance was increased in the pre‐nest‐building stage compared to the post‐nest‐building stage of green‐backed tits. This suggests that the microbiological environment of the nest may have changed in the post‐nest‐building stage of green‐backed tits, leading to an increase in beneficial bacteria and a decrease in pathogenic bacteria in the nest.

At the genus level, LEfSe results showed that the relative abundance of several dominant bacteria, *Pseudonocardia*, *Actinomycetospora*, *Nocardioides*, *Rhodococcus*, *Stenotrophomonas*, and *Pantoea*, differed significantly between the pre‐nest‐building and post‐nest‐building stages of green‐backed tits (Figure 6). Nests in the post‐nest‐building stage of green‐backed tits were dominated by beneficial bacteria, such as the high proportion of *Pseudonocardia* (6.19%), *Actinomycetospora* (3.73%) and *Nocardioides* (2.70%) all belonging to the Actinomycetota. Actinomycetota is a common microbiota phylum with antimicrobial properties and can control pests and diseases through the efficient bactericidal action of their metabolites (Pongen et al. 2023). Opportunistic pathogens were mainly present in the pre‐nest‐building stage of green‐backed tits, such as *Rhodococcus* (4.09%), *Stenotrophomonas* (1.66%) and *Pantoea* (1.01%). These pathogens cause multiple forms of infection, including respiratory tract, lung, and wound infections, which can be fatal for infants, post‐operative, and immunocompromised patients (Brooke 2012; Ranganath et al. 2024; Ruan et al. 2022). Further comparative analysis demonstrated a significant enrichment of beneficial bacterial taxa, such as *Pseudonocardia*, within the nest microbiota during the post‐nest‐building stage relative to the pre‐nest‐building stage of green‐backed tits (*p* < 0.05). Conversely, pathogenic bacteria like *Rhodococcus* were significantly less prevalent in the post‐nest‐building stage of green‐backed tits (*p* < 0.05) (Figure 5). Therefore, our study concluded that the bacterial composition had significant changes in the post‐nest‐building stage of green‐backed tits. The nest microbiota in the post‐nest‐building stage, compared to the pre‐nest‐building stage of green‐backed tits, was primarily dominated by beneficial bacteria, demonstrating an overall trend of increased beneficial bacteria and a decrease in potentially pathogenic bacteria.

### 3.2 Changes in the Fungal Composition in the Post‐Nest‐Building Stage of Green‐Backed Tits

For birds, fungi are opportunistic pathogens, and most fungal infections are caused by a weakened immune system or malnutrition (Ogórek et al. 2022). In our study, the diversity of fungal communities was significantly higher in the post‐nest‐building stage than in the pre‐nest‐building stage of green‐backed tits (*p* < 0.001). At the phylum level, the fungal composition in the pre‐ and post‐nest‐building stages of green‐backed tits showed no significant differences, with both primarily consisting of Ascomycota and Basidiomycota. These phyla are not only the most widely distributed and diverse groups of fungi, but also major plant pathogens (Doehlemann et al. 2017). The Ascomycota phylum includes yeasts, filamentous fungi, and lichenized fungi (Bhunjun et al. 2021). Many of these fungi produce mycotoxins, which will cause severe acute poisoning in humans and animals, potentially leading to death (Gurikar et al. 2023). The observed reduction in the abundance of Ascomycota in the nest may be attributed to the suppression of pathogenic fungi in the post‐nest‐building stage of green‐backed tits.

At the genus level, LEfSe analysis revealed that the relative abundance of several dominant fungal genera, including *Arcuadendron*, *Cyphellophora*, *Penicillium*, and *Xenochalara*, differed significantly between the pre‐ and post‐nest‐building stages of green‐backed tits (Figure 6). Several genera with high abundance in the pre‐nest‐building stage were essentially potential pathogenic fungi. For example, the most abundant genus, *Penicillium*, is a fungus that produces a wide range of mycotoxins and can be found in a variety of habitats (Visagie et al. 2016). *Penicillium griseofulvum* has been found in captive toucanets (Ramphastidae) in Finland and can cause avian penicilliosis, which invades the lungs, gas vesicles, liver, and other tissues of toucanets, resulting in the death of the toucanet (Aho et al. 1990). In the pre‐nest‐building stage of green‐backed tits, another pathogen with a relatively high abundance is *Aspergillus*, which includes two common fungi: the extremely virulent *Aspergillus flavus* and *Aspergillus fumigatus* , which are potentially pathogenic for homoiothermous organisms (Kornillowicz‐Kowalska and Kitowski 2018). *Aspergillus* is a common pathogen in birds and is considered one of the most threatening opportunistic pathogens. It not only causes fungal infections in the lungs and gas vesicles of birds (Barathidasan et al. 2013) but also penetrates eggshells and egg membranes, causing the death of unhatched embryos (Ogórek et al. 2022). *Aspergillus* can be found in the nests and feathers of many wild birds, such as marsh harrier ( *Circus aeruginosus* ) (Kornillowicz‐Kowalska and Kitowski 2018), gray heron ( *Ardea cinerea* ) (Ogórek et al. 2022) and woodpeckers (Shi et al. 2024). *Penicillium* and *Aspergillus* showed large reductions in abundance within post‐nest‐building by green‐backed tits, indicating that the nest‐building behavior of green‐backed tits inhibits the growth of these pathogenic fungi. However, it is noteworthy that the abundance of *Cladosporium* increased in the post‐nest‐building stage of green‐backed tits. *Cladosporium* is a ubiquitous environmental fungus, and its spores have been reported to trigger asthma and allergies, posing a potential threat to the health of both adult green‐backed tits and their chicks (Kanj et al. 2023; Lee et al. 2025). Overall, a clear trend of reduced pathogenic fungi was observed in the post‐nest‐building stage of green‐backed tits, indicating that the nest‐building behavior of green‐backed tits significantly influenced the fungal composition within the nests.

### 3.3 Changes in the Microbial Network Tits During the Post‐Nest‐Building Stage of Green‐Backed Tits

In terms of microbial interactions, microbiota engage in multifaceted and complex relationships, ranging from symbiosis to competition, as they adapt to environmental changes (Hibbing et al. 2010). Although co‐occurrence network analyses cannot fully show the associations between microbiota, they can help us understand how microbiota will respond to environmental changes. Therefore, based on co‐occurrence network analysis, we investigated the response of the microbial community within the nest to the nest‐building behavior of the green‐backed tit. The results indicated that the green‐backed tits reduced the complexity of the microbial network in the post‐nest‐building stage. In co‐occurrence networks, the complexity of the network can be characterized by several topological metrics, including density, average degree, and clustering coefficient (Li et al. 2024; Wu et al. 2024). The reduction of these metrics suggests a marked simplification of the microbial network architecture in the post‐nest‐building stage of green‐backed tits. Therefore, we hypothesize that this results from active modifications of nest microbiota in the post‐nesting stage of green‐backed tits. To enhance offspring fitness and survival, these birds may selectively promote beneficial microorganisms while suppressing potential pathogens, thereby restructuring the microbial community into a more beneficial composition and reducing the complexity of microbial interactions within the nest environment.

Our study further revealed that the green‐backed tits enhance the stability of the nest microbial network in the post‐nest‐building stage. This community stability can be predicted by the proportion of positively and negatively associated relationships between taxa (Hernandez et al. 2021). A positive correlation typically reflects a symbiotic relationship or a parasitism relationship between microbial communities, whereas a negative correlation often indicates predatory or competitive relationships among these communities (Wang et al. 2021). Relevant studies have shown that the relative proportion of positive and negative correlations will influence community stability. An increase in the dominance of positive correlations or a decrease in the dominance of negative correlations may individually undermine community stability (Mougi and Kondoh 2012; Coyte et al. 2015; Suweis et al. 2014). In our study, the increase in negative correlations within the microbial networks in the post‐nest‐building stage of green‐backed tits suggests enhanced stability of the nest's microbial community. This increased stability likely improves the network's resistance to external environmental perturbations (Wu et al. 2021; Chen et al. 2025; Gong et al. 2024; Yuan et al. 2025).

Some studies have shown that diverse ecosystems maintain stability by reducing complexity (Wu et al. 2024; Hu et al. 2022; Yonatan et al. 2022). In ecosystems with low complexity, species interactions are generally weak, and the presence or absence of any single species tends to have minimal impact on the dynamics of other species within the community. In highly complex ecosystems, where species are more closely related and have strong interactions, an increase or decrease in one species can have a serious impact on other species (Wu et al. 2024). Our findings indicate that in the post‐nest‐building stage of green‐backed tits, the complexity of the microbial network decreased, while the stability of the network increased.

### 3.4 Factors Causing Changes in the Microbial Community Within Nests

The changes in the microbial community within the nest are driven by multiple factors, among which the characteristics of nesting materials and the contact transmission by parent birds are the two core factors. Birds show significant preferences for nesting materials, and this selection directly affects the composition of the microbial community in the nest. Take tits as an example; different species exhibit a high degree of selectivity for the types of bryophytes used for nesting (Rydgren et al. 2023; Wesołowski and Wierzcholska 2018). Bryophytes play a key role in regulating microbial communities due to their unique chemical properties (such as antibacterial, antiviral, antifungal, anticancer, and insecticidal activities) (Asakawa and Ludwiczuk 2017). When crushed, bryophytes release a strong odor that acts as a natural pest repellent, and the various bioactive secondary metabolites they produce further stabilize the microbial environment in the nest, forming a relatively healthy microbial structure (Stelmasiewicz et al. 2023; Commisso et al. 2021). In addition, some tit species actively incorporate feathers into their nests. The antibacterial properties of feathers make their use regarded as a potential self‐therapeutic strategy (Ruiz‐Castellano et al. 2019), which further confirms the targeting of material selection in microbial regulation.

The frequent contact between parent birds and nest materials reshapes the microbial community through microbial transmission and bidirectional sharing. Studies have shown that there is a significant similarity between avian‐associated microbial communities (microbes on the skin, cloaca, and feathers) and nest‐associated microbial communities, and the higher the frequency or intensity of contact, the more pronounced this similarity becomes (Chen et al. 2020; Diez‐Méndez et al. 2023; van Veelen et al. 2017; Goodenough et al. 2017). As proposed by Goodenough et al. (Goodenough et al. 2017), there is bidirectional microbial sharing between birds and their nests, leading to the homogenization of their microbial communities: common genera from bird intestines or skin (such as *Enterococcus* and *Staphylococcus*) can be detected in nest materials, while typical plant or soil microbes (such as *Acrodontium*, *Beauveria*, and *Gracilibacillus*) are found on bird feathers and skin (Goodenough et al. 2017). Research confirms that avian uropygial glands harbor bacteria capable of producing antimicrobial compounds (Bodawatta et al. 2020; Soler et al. 2024). During preening, these microbes are spread on the surface of feathers along with gland secretions, and then colonize the nest's microenvironment through physical friction between parent birds and nest materials, effectively inhibiting the proliferation of pathogenic bacteria. Moreover, this antibacterial activity is significantly stronger during the nesting period compared to the non‐nesting period (Soler et al. 2024). Meanwhile, functional antibacterial bacteria in the feces of parent birds can be vertically transmitted through the coprophagy behavior of nestlings, accelerating the maturation of nestlings' gut microbiota, increasing microbial diversity, and reducing the number of pathogenic bacteria (Videvall et al. 2023).

In this study, after the green‐backed tits built their nests, the microbial community in the nest showed clear positive changes: the abundance of beneficial bacteria increased, the number of pathogenic bacteria decreased, and the complexity of the microbial symbiotic network was reduced while its stability was enhanced. These phenomena precisely indicate that birds actively modify the microenvironment in the nest to create stable and healthy growth conditions for their offspring. The complex microbial dynamics in the nest stem from both the active introduction of specific nesting materials by parent birds and the passive exchange resulting from frequent contact between parent birds and the nest; together, these two factors shape the critical microenvironment on which the growth and development of nestlings depend. Although this study has not yet fully clarified the specific proportion of the contribution of microbial transfer by parent birds to the nest's microenvironment, the active and selective use of nesting materials with specific antibacterial properties, such as bryophytes, by green‐backed tits strongly suggests that this is an adaptive behavior through which they actively regulate the microbial community in the nest by selecting materials.

## 4 Conclusions

In this study, we explored the effects of green‐backed tits nesting on the microbial community within the nest. Our findings were that: (1) Compared with the pre‐nest‐building stage of green‐backed tits, the microbial composition changed within the post‐nest‐building stage of green‐backed tits, with the nest showing an overall increase in beneficial microorganisms and a decrease in potentially pathogenic microorganisms; (2) reduced complexity and increased stability of microbial networks in the post‐nest‐building stage of green‐backed tits. Overall, we have confirmed that the green‐backed tits altered the nest's microecology in the post‐nest‐building stage of green‐backed tits. This change is likely aimed at ensuring the smooth progression of the breeding process and the normal development of their offspring; the green‐backed tits provided a healthy and stable microenvironment for their growth. By conducting research on the microecology within the nests of green‐backed tits, we hope to gain a better understanding of the complex relationships between birds and the microorganisms in their nests.

## 5 Materials and Methods

### 5.1 Sample Collection

From March to June 2024, green‐backed tits were attracted to build nests by hanging artificial nest boxes in Aha Lake National Wetland Park (altitude 1100~1350 m, 106°36′59″~106°40′44″ E 26°30′40″~26°33′55″ N, in Guiyang, Guizhou Province, China). Field observations have recorded that the breeding season of the green‐backed tits generally spans from March to June. Nest‐building begins progressively in March and is completed by early April, after which egg‐laying commences. The incubation period typically lasts 14–15 days, with a clutch size predominantly ranging from 4 to 7 eggs. According to the breeding habits of the green‐backed tits, we hang nest boxes 3 months before the breeding season. The dimensions of the nest boxes were 13 cm × 12 cm × 27 cm, with 1.5 cm thick panels and a 4.0 cm diameter entrance opening. All nest boxes were placed in woodland areas with uniform and continuous environments and less human disturbance. The distance between any two nest boxes was > 50 m and was randomly distributed. In the early stage of breeding, the frequency of inspections was maintained at weekly intervals, changing to every 2 days after the discovery of nest material. The nesting materials of green‐backed tits consist of bryophytes, hair, and grass stems. In this study, we defined the period characterized by completed nest materials but prior to egg‐laying as the post‐nest‐building stage. Nest boxes that showed no evidence of nest predation were selected as potential study subjects. The period before nest construction, when nest boxes were empty (around late February), was defined as the pre‐nest‐building stage. From the pool of eligible post‐nest‐building nest boxes, we randomly selected 9 for microbial sampling. Corresponding control samples were collected from the same nest boxes during the pre‐nest‐building stage. A total of 18 microbial samples were collected from post‐nest‐building (*N* = 9) and pre‐nest‐building (*N* = 9) nest boxes. We collected microbial samples by wiping the edges of the nest boxes for 10 s using a sterile cotton swab (previously moistened with sterile water) and then passing it across the bottom in a cross pattern (Xin et al. 2023; Goodenough et al. 2017). Finally, it was transferred to a −80°C ultra‐low temperature refrigerator for freezing and preservation.

### 5.2 DNA Extraction and PCR Amplification

Microbial sample DNA was extracted using FastDNA Spin Kit for Soil (MP Biomedicals, USA) and the quality of extracted genomic DNA was checked using 1% agarose gel electrophoresis. DNA purity and concentration were measured using a NanoDrop2000 spectrophotometer. PCR amplification of the target fragment of the V3‐V4 region of the bacterial 16S rRNA gene was performed using bacterial universal primers 799F (5′‐AACMGGATTAGATACCCKG‐3′) and 1193R (5′‐ACGTCATCCCCACCTTCC‐3′), and amplification of the internal transcribed spacer region (ITS) was performed using fungal universal primers ITS1F (5′‐CTTGGTCATTTAGAGGAAGTAA‐3′) and ITS2R (5′‐GCTGCGTTCTTCATCGATGC‐3′). The PCR reaction mixture (20 μL total volume) contained: 10 μL of 2× Pro Taq Master Mix, 0.8 μL of each primer (5 μM), 10 ng of template DNA, and ddH₂O to volume. Amplification conditions consisted of initial denaturation at 95°C for 3 min; 27 cycles of 95°C for 30 s, 55°C for 30 s, and 72°C for 45 s; followed by a final extension at 72°C for 10 min and holding at 4°C. The obtained amplification products were electrophoresed, purified, and quantified and then subjected to paired‐end sequencing using the Illumina PE300 sequencing platform of Majorbio Bio‐pharm Technology Co. Ltd. (Shanghai, China). Sequencing yielded paired‐end (PE) sequence data. Paired reads were merged using FLASH (Magoč and Salzberg 2011), and quality control filtering for both read quality and merging efficiency was performed using fastp (Chen et al. 2018). Finally, we obtained optimized sequences. The optimized sequences were subjected to extraction of non‐repetitive sequences, with single unique sequences (those without duplicates) removed. UPARSE was used to cluster the non‐repetitive sequences (excluding single sequences) into OTUs at a 97% similarity threshold, with chimeric sequences filtered out during the clustering process (Edgar 2013). Taxonomy assignment and removal of non‐bacterial sequences were performed against the SILVA database (Pruesse et al. 2007), and against the UNITE database to remove non‐fungal sequences (Kõljalg et al. 2013).

### 5.3 Data Analysis

Based on the OTUs information, alpha diversity indices including Chao1, Ace (reflecting community richness), Shannon, and Simpson (reflecting community diversity) were calculated with Mothur v1.30.1. (Schloss et al. 2009). The Paired Samples t‐tests were used to test whether there were significant differences in microbial diversity within nests between the post‐ and pre‐nest‐building. Beta diversity analysis was performed based on the Bray‐Curtis distance algorithm to visualize the degree of similarity or difference in the composition of microbial communities in the samples by principal coordinates analysis (PCoA), and analysis of similarities (ANOSIM) was used to test differences between groups. Linear Discriminant Analysis (LDA) effect size (LEfSe) was performed to identify the significantly abundant taxa (phylum to genera) across groups (Segata et al. 2011). Networks were utilized to explore the co‐occurrence patterns of bacterial and fungal taxa in the post‐ and pre‐nest‐building of green‐backed tits. We constructed Spearman correlation analyses (*r* > 0.7 or r < −0.7, *p* < 0.01) to explore the interactions between genera. Calculating networks topology characteristics parameters with the Networkx V1.11 Package (Hagberg et al. 2008) and the network visualization and property measurements in the Gephi V 0.10.1 software (Bastian et al. 2009).

## Author Contributions

**Nan Yang:** conceptualization (equal), formal analysis (lead), investigation (equal), methodology (equal), software (lead), validation (lead), visualization (lead), writing – original draft (lead), writing – review and editing (equal). **Jiajia Xin:** conceptualization (equal), data curation (equal), formal analysis (equal), investigation (equal), methodology (equal), writing – original draft (equal). **Qun Tu:** formal analysis (equal), methodology (equal). **Xiaoyang Bao:** formal analysis (equal), methodology (equal). **Haibo Zhang:** investigation (equal). **Yijiang Su:** investigation (equal). **Xiongwei Yang:** investigation (equal). **Canshi Hu:** conceptualization (equal), formal analysis (supporting), funding acquisition (lead), methodology (equal), resources (equal), supervision (equal), writing – review and editing (equal).

## Ethics Statement

The present study was conducted without direct contact with birds, ensuring no harm was caused to the avian subjects. The experimental procedures were reviewed and approved by the Experimental Animal Ethics Subcommittee of Guizhou University (Approval No. EAE‐GZU‐2023‐E006).

## Conflicts of Interest

The authors declare no conflicts of interest.

## Data Availability

Metagenomic reads produced in this study are available under NCBI BioProject accession number PRJNA1224938 and PRJNA1224922.
